# Effects of Aged Garlic Extract on Cholinergic, Glutamatergic and GABAergic Systems with Regard to Cognitive Impairment in Aβ-Induced Rats

**DOI:** 10.3390/nu9070686

**Published:** 2017-07-01

**Authors:** Piyaporn Thorajak, Wanassanun Pannangrong, Jariya Umka Welbat, Wunnee Chaijaroonkhanarak, Kittisak Sripanidkulchai, Bungorn Sripanidkulchai

**Affiliations:** 1 Department of Anatomy, Faculty of Medicine, Khon Kaen University, Khon Kaen 40002, Thailand; piyaporn.anatomy@gmail.com (P.T.); wankun@kku.ac.th (W.P.); jariya@kku.ac.th (J.U.W.); cwunnee@kku.ac.th (W.C.); skitti@kku.ac.th (K.S.); 2 Center for Research and Development of Herbal Health Products, Khon Kaen University, Khon Kaen 40002, Thailand; 3 Neuroscience Research and Development Group, Khon Kaen University, Khon Kaen 40002, Thailand

**Keywords:** aged garlic extract, Alzheimer’s disease, amyloid-β, cholinergic neurons, glutamate decarboxylase, radial arm maze, vesicular glutamate transporters

## Abstract

Alzheimer’s disease (AD) has been linked to the degeneration of central cholinergic and glutamatergic transmission, which correlates with progressive memory loss and the accumulation of amyloid-β (Aβ). It has been claimed that aged garlic extract (AGE) has a beneficial effect in preventing neurodegeneration in AD. Therefore, the objective of this study was to investigate the effects of AGE on Aβ-induced cognitive dysfunction with a biochemical basis in the cholinergic, glutamatergic, and GABAergic systems in rats. Adult male Wistar rats were orally administered three doses of AGE (125, 250, and 500 mg/kg) daily for 65 days. At day 56, they were injected with 1 μL of aggregated Aβ (1–42) into each lateral ventricle, bilaterally. After six days of Aβ injection, the rats’ working and reference memory was tested using a radial arm maze. The rats were then euthanized to investigate any changes to the cholinergic neurons, vesicular glutamate transporter 1 and 2 proteins (VGLUT1 and VGLUT2), and glutamate decarboxylase (GAD) in the hippocampus. The results showed that AGE significantly improved the working memory and tended to improve the reference memory in cognitively-impaired rats. In addition, AGE significantly ameliorated the loss of cholinergic neurons and increased the VGLUT1 and GAD levels in the hippocampus of rat brains with Aβ-induced toxicity. In contrast, the VGLUT2 protein levels did not change in any of the treated groups. We concluded that AGE was able to attenuate the impairment of working memory via the modification of cholinergic neurons, VGLUT1, and GAD in the hippocampus of Aβ-induced rats.

## 1. Introduction 

Memory formation is a complex process that is associated with various neurotransmitter systems. Three major systems, including the cholinergic, glutamatergic, and GABAergic, are commonly involved and have predominate roles in the process [1,2,3,4]. Acetylcholine (ACh), a neurotransmitter of the cholinergic system, released within the hippocampal circuits is important for learning and memory. It is a powerful presynaptic modulator of both glutamatergic and GABAergic synaptic transmission [1]. Ach is synthesised by the Choline acetyltransferase (ChAT) enzyme which is found in high concentrations in cholinergic neurons as visualized by choline acetyltransferase (ChAT) immunohistochemcal staining. In the modulation of network activity, Ach-activated muscarinic acetylcholine receptors either directly [3], or through associative interactions with glutamatergic synaptic inputs, promote longer-term synaptic plasticity [5]. Glutamate, a neurotransmitter of the glutamatergic system, acts as an excitatory transmitter in the major pathways of hippocampal formation. The quantal release of glutamate depends on its transport into synaptic vesicles [6]. Two vesicular glutamate transporters, vesicular glutamate transporters 1 (VGLUT1) and vesicular glutamate transporters 2 (VGLUT2), are the predominant isoforms of the excitatory glutamatergic terminals in the brain. Both transporters mediate glutamate uptake into the synaptic vesicles of glutamatergic neurons [7,8,9]. The GABAergic system provides reciprocal presynaptic inhibition of cholinergic and glutamatergic inputs through the hippocampus formation activation of GABA_B_ receptors [10]. GABA is synthesized via the catalytic activity of glutamic acid decarboxylase (GAD) enzymes. Two molecular forms of GAD, expressed in the brain, are GAD 65 and GAD 67 [11]. 

The hippocampus is associated with spatial learning and memory [12], in which the integration of cognitive and the normal function in glutamatergic and cholinergic systems is necessary for spatial learning [13]. Glutamate and GABA are the major excitatory and inhibitory neurotransmitters in the central nervous system. Integration of excitatory and inhibitory signals is a basic function of neuronal communication [14]. Thus, plasticity of synapses related to learning and memory requires adequate levels of excitation and inhibition to be maintained [15]. Thus, the alteration properties of hippocampal neurons and/or dysfunction of three neurotransmitters are involved in cognitive impairments were found in Alzheimer’s disease (AD) [3,16,17,18]. 

Amyloid-β (Aβ) is known as a major cause of cognitive impairment. Several studies used Aβ to investigate the presence of cognitive disability in rat models as observed by behavioural tests [19,20,21]. Aβ plaques in the cerebral cortex and hippocampus may relate to the intellectual decline in AD [22]. Previous studies had reported that the neurotoxicity of Aβ peptides involved many neuronal events, such as the deficiency of neurotransmitters, the decrease in vesicular transporters, and synaptic transmissions, leading to neuronal cell death [23,24,25,26]. There have been several studies on the neuroprotective effects of natural products in AD models. Several compounds from various plants may have properties that benefit the cholinergic, glutamatergic, and/or GABAergic systems. This information may aid in the study of neuroprotective and cognitive improvement leading to preventative and therapeutic strategies against AD. 

Aged garlic extract (AGE) is a garlic (*Allium sativum* L.) product that has been reported to be beneficial in preventing neurodegeneration because of its antioxidant and Aβ-lowering properties [26]. Long-term administration of AGE has been reported to prevent atrophic changes in the forebrain and learning deficits in senescence-accelerated mice [27], as well as to improve short-term recognition memory in Aβ-induced rats [28]. In addition, adding AGE to a culture medium has been shown to result in a concentration-dependent increase in the survival and axonal branching of cultured rat hippocampal neurons [29]. However, there is a lack of direct evidence regarding the role of AGE on learning and memory associated with the cholinergic, glutamatergic, and GABAergic systems. Therefore, the present study was undertaken to investigate the beneficial effects of AGE on Aβ-induced cognitive dysfunction with a biochemical basis in the cholinergic, glutamatergic, and GABAergic systems in rats.

## 2. Materials and Methods 

### 2.1. Preparation of Aged Garlic Extract 

AGE was provided by the Center for Research and Development of Herbal Health Products (CRD-HHP) at Khon Kaen University in Khon Kaen, Thailand. AGE was prepared by soaking chopped garlic (obtained from Srisaket province in Thailand) in 30% ethanol at a 1:3 ratio for approximately 15 months under light protection at room temperature. After filtration, ethanol in the filtrate was removed using a rotary evaporator, leaving dried AGE powder with a percent yield of 3.8. HPLC analysis of AGE revealed the bioactive compounds to be *S*-allylcysteine (SAC) and allicin at concentrations of 30.96 mg/g and 32 μg/g, respectively (Petty patent No. 3506, Thailand). The AGE was dissolved in distilled water at concentrations of 125, 250, and 500 mg/kg BW before being administered to the rats.

### 2.2. Animals and Treatment Protocol

Forty adult male Wistar rats (180–220 g) were purchased from the National Laboratory Animal Center at Mahidol University in Nakhon Pathom, Thailand. With free access to water and standard pellet food (CPF, Saraburi, Thailand), the rats were maintained on a 12 h light/12 h dark cycle at 23 ± 2 °C at the Northeast Laboratory Animal Center at Khon Kaen University (Thailand). All studies were carried out in accordance with the National Institute of Health (NIH) Guide for the Care and Use of Laboratory Animals, and the experimental design was approved by the Khon Kaen University Institutional Animal Ethics Committee (AEKKU 88/2555). After one week of acclimatization, the animals were divided into five groups, with each group consisting of eight rats. The groups were as follows:

Group 1 (Vehicle control) received distilled water as a vehicle. 

Group 2 (Vehicle + Aβ) received distilled water and were injected with Aβ (1–42) into the lateral ventricle. 

Group 3 (AGE125 + Aβ) received AGE (125 mg/kg BW, per oral) and were injected with Aβ (1–42) into the lateral ventricle. 

Group 4 (AGE250 + Aβ) received AGE (250 mg/kg BW, per oral) and were injected with Aβ (1–42) into the lateral ventricle. 

Group 5 (AGE500 + Aβ) received AGE (500 mg/kg BW, per oral) and were injected with Aβ (1–42) into the lateral ventricle. 

**Figure 1:**
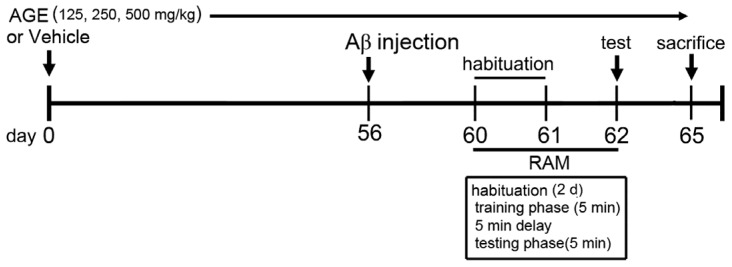
Schematic diagram of drug treatment and behavioural tests. Rats were injected with Aβ (1–42) into both sides of the lateral ventricle after 56 days of drug treatments. (RAM: radial arm maze test, Aβ: amyloid-β (1–42)).

Vehicle and AGE solution were orally administered once daily for sixty-five consecutive days. At day 56, the rats in groups 2–5 were bilaterally injected with 1 μL of Aβ (1–42) into each side of both lateral ventricles. At day 62, all rats were tested for working and reference memory performance using a radial arm maze test (RAM) (Figure 1). At day 65, all rats were anesthetized with an overdose of thiopental sodium (Jagsonpal pharmaceutical Ltd., New Delhi, India) via injection and were transcardially perfused with 0.9% normal saline solution.

### 2.3. Aβ (1–42) Treatment

Aβ (1–42) peptide (Alexis Biochemicals, San Diego, CA, USA) was dissolved in glacial acetic acid at a concentration of 1 mg/mL and incubated at 37 °C for 24 h to cause peptide aggregation. The rats were anesthetized with thiopental sodium (35 mg/kg BW, intraperitoneal) and then injected with 1 μL of aggregated Aβ (1–42) peptide bilaterally into the lateral ventricle at a rate of 0.2 μL/min, with stereotaxic coordinates of anterior-posterior (AP)-0.8 mm from the bregma, lateral (L) ±1.5 mm from the midline, and superior-inferior (SI)-3.8 mm from the dura [28]. After injection, the needle was slowly removed. The rats were kept on a warm pad (32–33 °C) and returned to their cages.

### 2.4. Radial Arm Maze Test (RAM)

**Figure 2:**
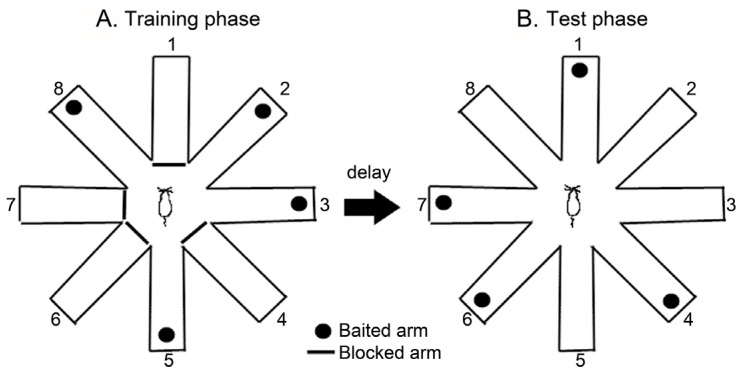
The schematic diagram of the eight-arm radial maze. The animals were tested in the RAM with a 5 min delay.

Working and reference memory were measured using an eight-arm radial maze (11 cm × 50 cm) extending radially from a central area (30 cm wide octagon). The maze was placed 50 cm above the floor and surrounded by various extra-maze visual cues located in the same positions throughout the study. A food cup was located at the distal end of each arm. Before the testing began, the rats were habituated to the maze environment for two consecutive days, after which subsequent performance tests were conducted. These trials consisted of a training phase and a test phase, separated by a delay. In the training phase, the four arms (arms 1, 4, 6, and 7) were randomly blocked, and the four remaining arms (arms 2, 3, 5, and 8) were baited (Figure 2A). Each rat was given 5 min. to enter the four open arms or retrieve the bait, after which it was returned to its home cage for the delay period. After a 5 min. delay, each rat was placed back into the maze for the test phase. During the test phase, all eight arms were opened. However, the bait was only in the arms that had previously been blocked (Figure 2B). The number of arm entries was recorded along with two types of errors: working memory errors (re-entries into an arm containing food or an arm not baited), and reference memory errors (entering an arm that does not contain the bait). The maze was cleaned with a 20% ethanol solution and dried with a cloth before the next animal was tested [30].

### 2.5. Tissue Preparation

After euthanasia, the whole brain of each rat was quickly removed, put on ice and cut at the midline dividing the brain into left and right hemispheres. The left hemisphere was cryopreserved in sucrose solution (30%) and fixed in ice cold 4% paraformaldehyde solution for immunohistochemical investigation of ChAT using a free-floating technique. The hippocampus was taken from the right hemisphere and homogenized immediately (10% *w*/*v* homogenate) in an ice-cold medium containing 20 mM Tris-HCl (pH 7.4) and 320 mM sucrose. The homogenate was centrifuged at 15,000× *g* at 4 °C for 10 min. The supernatant was used for Western blot analysis of GAD, VGLUT1, and VGLUT2.

### 2.6. Immunohistochemistry 

Immunohistochemical staining for the ChAT enzyme was performed on serial and coronal sections of the entire brain using the free-floating technique. The 35 µm-thick sections were cut using a sliding microtome (Thermo, Walldorf, Germany) and put in cold 0.01 M phosphate-buffered saline (PBS), pH 7.4. The first series of immunohistochemistry analyses of the ChAT enzyme was performed on free-floating sections at 4 °C. Sections were then incubated in blocking serum (1% bovine serum albumin in PBS) for 1 h and further incubated overnight with a polyclonal antibody for ChAT (1:100, Merck Millipore, Darmstadt, Hesse, Germany) in blocking serum at 4 °C. Then, the sections were incubated with peroxidase conjugated secondary antibody (1:1000, Invitrogen, Carlsbad, CA, USA) for 2 h. After being washed in PBS, the sections were treated with 0.001% of diaminobenzidine tetrahydrochloride dihydrate (Sigma, St. Louis, MO, USA) in PBS containing 0.003% H_2_O_2_ (Merck Millipore, Darmstadt, Hesse, Germany). The sections were then mounted on gelatin-coated glass slides, allowed to dry overnight, counterstained with cresyl violet, dehydrated and cover-slipped under DPX (Sigma, St. Louis, MO, USA). All slides were examined under a light microscope (Nikon Microscope ECLIPSE E200 MVR, Nikon Corp, Tokyo, Japan) at 40× objective. A Prosilica GT digital camera (Dynatech Inst, Bangkok, Thailand) connected to a computer was mounted on top of the microscope. Every forth section throughout the entire rostrocaudal extent of the hippocampus of each brain was selected for a blind count of ChAT-immunoreactive cells in the hippocampus. The raw data were multiplied by four to achieve an estimate of the total number of ChAT-immunoreactive cells in each hippocampus sample. The number of ChAT-immunoreactive cells was analysed using the method modification described by Huang and Herbert (2006) [31].

### 2.7. Western Blot Analysis

Hippocampal tissue was prepared for Western blotting as has been previously described [32]. The protein concentrations (10 μg per lane, VGLUT1; 50 μg per lane, VGLUT2; 30 μg per lane, GAD) were separated by 10% SDS-PAGE (Bio-Rad Laboratories GmbH, Munich, Germany), transferred to a nitrocellulose membrane and blocked with 5% skim milk (Sigma, St. Louis, MO, USA) in 25 mM Tris-buffer saline containing 0.1% Tween 20 (TBS-T) at room temperature for 1 h. The membranes were then separately incubated with primary antibodies, including anti-VGLUT1 antibody (1:100, Abcam, Milton, Cambridge, UK), anti-VGLUT2 antibody (1:1000, Abcam, Milton, Cambridge, UK), anti-GAD 65 and 67 antibodies (1:1000, Merck Millipore, Darmstadt, Hesse, Germany), and GADPH as a reference protein (1:20,000, Abcam, Milton, Cambridge, UK), in TBS-T at 4 °C for 24 h. After extensive washing with TBS-T, the membranes were incubated with peroxidase-conjugated secondary antibody (1:1000, Merck Millipore, Darmstadt, Hesse, Germany) at room temperature for 2 h. Signals were visualized using a chemiluminescence substrate (Thermo Scientific, Waltham, MA, USA) and exposed onto film. The optical density of the bands was calculated after background subtraction using Image J analysis software (Windows version, National Institutes of Health, Bethesda, MD, USA).

### 2.8. Statistical Analysis

All statistical parameters were calculated using GraphPad Prism V 5.0 (GraphPad Software, LaJolla, CA, USA). The data are expressed as means ± standard error of mean (S.E.M.) of variance (ANOVA). This was followed by a post hoc Bonferoni test. A probability level of less than 0.05 was accepted as significant.

## 3. Results

### 3.1. Effect of AGE on Working and Reference Memory in Radial Arm Maze Test

**Figure 3:**
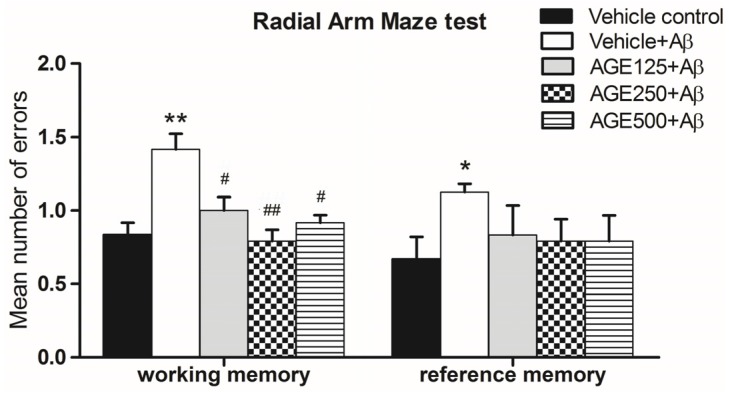
Number of working memory errors and reference memory errors made while looking for the baited arm in the RAM on day 6 after Aβ injection. Data are presented as mean ± S.E.M. (*n* = 8), *, ** = significant differences from the vehicle control group at *p* < 0.05 and 0.01, respectively; **^#^**,**^##^** = significant differences from the vehicle + Aβ group at *p* < 0.05 and 0.01, respectively.

As shown in Figure 3, Aβ-treated rats showed significant deficits in terms of the mean number of errors in working and reference memory (*p* < 0.01 and 0.05, respectively). When compared to the vehicle plus Aβ groups, the mean number of errors in groups that received AGE at any dose indicated that AGE significantly prevented working memory loss (One way ANOVA test, *F*_4, 35_ = 17.23, *p* < 0.05 and *p* < 0.01). There were also decreases in the mean number of errors related to reference memory, but this difference was not statistically significant (one-way ANOVA test, *F*_4, 35_ = 2.93, *p* > 0.05). These results indicate that exposure to AGE could improve working memory as demonstrated through the use of the RAM.

### 3.2. Effect of AGE on Cholinergic Neurons in the Hippocampal Region 

**Figure 4:**
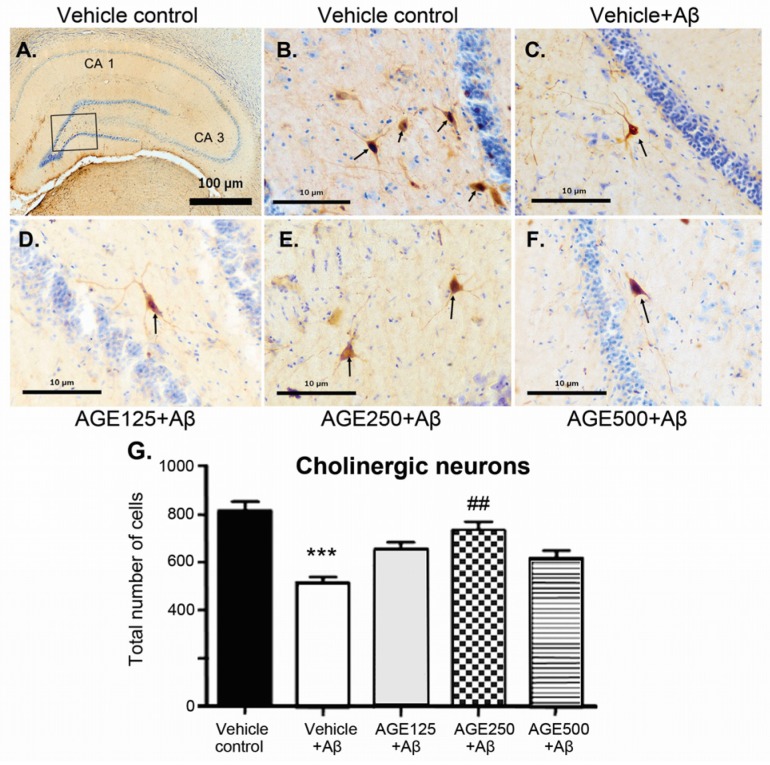
The neuroprotective effect of AGE (aged garlic extract) on cholinergic neurons in the hippocampal region of Aβ-induced rats. (**A**–**F**) represent the photomicrographs of brain section showing the distribution of cholinergic neurons by double staining of Nissl stain with cresyl violet and immunohistochemistry stained with polyclonal ChAT (choline acetyltransferase) antibodies in the vehicle control group (**A** and **B**), vehicle + Aβ (**C**), AGE125 + Aβ (**D**), AGE250 + Aβ (**E**) and AGE 500 + Aβ (**F**). (**G**) represents the number of ChAT neurons. The cholinergic neurons are indicated with arrows. Data are presented as mean ± S.E.M. (*n* = 8), *** = significant differences from the vehicle control group at *p* < 0.001 and **^##^** = significant differences from the vehicle + Aβ group at *p* < 0.01.

Immunohistochemical study performed with the ChAT antibody showed long positive fibres of pyramidal-like cells throughout the hippocampal formation in all groups (Figure 4). Choline acetyltransferase-immunoreactive cells were relatively large and were observed in all layers of the hippocampus. When compared to the vehicle control group, the density of ChAT-immunoreactive cells in all regions of the hippocampus of the vehicle plus Aβ group was significantly lower (*p* < 0.001). Although all doses of AGE exhibited a neuroprotective effect against Aβ-induction neurotoxicity, only AGE at a dose of 250 mg/kg BW significantly restored the density of cholinergic neurons in the hippocampus (*p* < 0.01) (Figure 4G).

### 3.3. Effect of AGE on VGLUT1, VGLUT2, and GAD 65 and 67 in the Hippocampal Region

**Figure 5:**
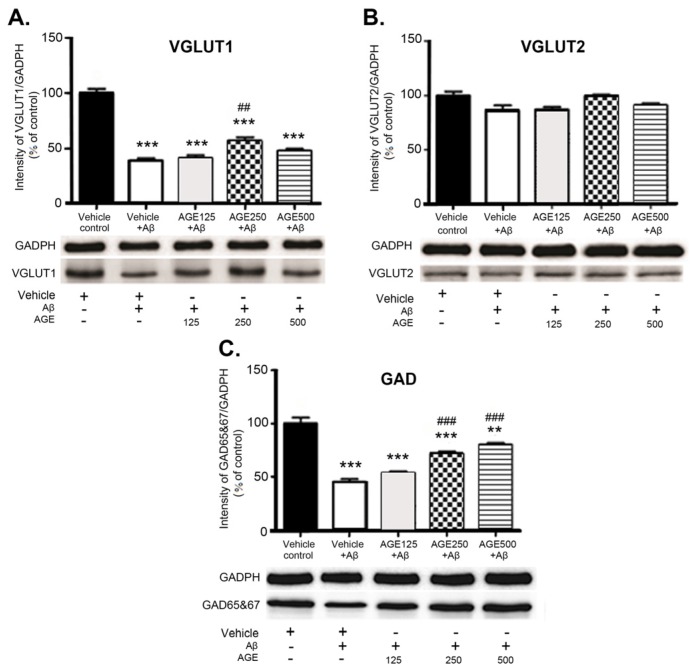
Effects of AGE on amounts of VGLUT1 (**A**), VGLUT2 (**B**) and GAD 65 and 67 (**C**) in the rat hippocampus. Data are presented as mean ± S.E.M (*n* = 8), **, *** = significant differences from the vehicle control group at *p* < 0.01 and 0.001 respectively **^##^**, **^###^** = significant differences from the vehicle + Aβ group at *p* < 0.01 and 0.001, respectively. VGLUT1 and VGLUT2, vesicular glutamate transporter 1 and 2 proteins; GAD, glutamate decarboxylase.

Western blot analysis showed the differentiated effect of AGE on levels of VGLUT1, VGLUT2, and GAD 65 and 67 in the hippocampal region of the rat brain. Aβ significantly reduced the levels of VGLUT1 and GAD 65 and 67 (*p* < 0.001) but did not affect VGLUT2 levels in the hippocampus (Figure 5). At a dose of 250 mg/kg BW AGE significantly increased the amount of VGLUT1 (*p* < 0.01) compared to vehicle plus Aβ. In contrast, AGE did not affect VGLUT2 levels in the hippocampal region at any dose. Treatment with medium and high doses of AGE markedly increased the amount of GAD 65 and 67 (*p* < 0.001) in the hippocampal region of the rat brain in a dose-dependent manner. 

## 4. Discussion

Degenerating neurons and synapses in the Alzheimer’s brain are located predominantly within susceptible regions including the hippocampus and cerebral cortex [33]. Biochemical investigations of brain tissue indicate that various neurotransmitters and modulators, including ACh, VGLUT and GABA, are differentially affected in various regions of Alzheimer’s brain [25,34,35]. The major component of senile plaques in the Alzheimer’s brain is the Aβ peptide. Neurons, glial cells, or synaptic markers can also be altered functionally and structurally by excessive Aβ levels [36,37]. The present study found that administration of Aβ (1–42) into the lateral ventricle caused significant decreases the number of ChAT-immunoreactive cells in the hippocampus (Figure 4). This is consistent with previous reports of Aβ oligomers inducing a major reduction in ChAT activity [38] and the lack of ChAT viability by attaching specifically to excitatory synapses, leading to cholinergic synapse dysfunction and cognitive impairment [39]. However, the persistence of cholinergic neurons in the hippocampus has been controversial and, according to some investigators, failed to be detected with immunohistochemical techniques [40]. Some studies reported the presence of ChAT-immunoreactive interneurons in the hippocampus [41,42,43,44]. Previous studies have reported that there are low numbers of ChAT-immunoreactive cells in the hippocampus. These cells are the interneurons, not the principal cells [44,45]. Although there is no clear role of these interneurons, Hefft et al. have shown that nicotinic acethylcholine receptors can be synaptically activated in rat hippocampal organotypic cultures [46]. However, the support on these findings still need further investigation. We also found that Aβ (1–42) caused predominant losses of glutamatergic and GABAergic markers in the hippocampus, as indicated by significant decreases in VGLUT1 and GAD densities (Figure 5). However, VGLUT2 density in the hippocampus of Aβ groups was not significantly different to those in the vehicle control. These findings are consistent with a previous report of decreases in the density of VGLUT1 and GAD [25,47], but not VGLUT2 in the hippocampus of Aβ-injected mice. Since VGLUT1 was found in the hippocampus more often than VGLUT2 [25], VGLUT2 is generally considered as a marker of glutamatergic neurons of thalamic origin. This may be the reason that Aβ toxicity does not result in changes in VGLUT2 in the hippocampus [48]. Moreover, Aβ (1–42), Aβ (31–35), and Aβ (34–39) toxicity was shown to decrease the number of GABAergic and glutaminergic neurons, suggesting that the degeneration of both neuronal groups is an indicator of early pathological changes in the brain [35]. Our results demonstrate that AGE exhibited a neuroprotective effect. Pretreatment of AGE at 250 mg/kg BW can prevent the decreases in the density of VGLUT1 and GAD in the hippocampus and protect from the loss of cholinergic neurons induced by Aβ (1–42). In terms of consumption, AGE at a dose of 250 mg/kg used in this study is equivalent to 6.5 g or 2–3 cloves of fresh garlic. These findings are in agreement with those of previous reports that AGE and SAC protected ChAT activity from reactive oxygen species (ROS) and mediated Aβ-induced damage in differentiated human cells. AGE treatment also reverses ROS-mediated declines in cholinergic function of neuronal cells by increasing levels of neuronal ChAT activity [26]. Treatment with AGE has been shown to restore the hypothalamic ChAT activity in thymectomized mice when compared to sham-operated controls [49]. However, the effects of AGE on glutamatergic and GABAergic systems was studied. These findings suggest that AGE can reverse the loss of cholinergic, glutamatergic, and GABAergic markers in cases of Aβ toxicity. This may result from AGE modifying cells’ defensive mechanisms. Furthermore, the chemical structure of organosulfur compounds present in the AGE, including SAC (major ingredient 30.96 mg/g), have allyl chains that could bind to the hydrophobic regions of Aβ and, thus, inhibit Aβ fibril formation [50]. Since adenosine and its degradative enzyme, adenosine deaminase, were reported to be modulated by garlic extract in the rat hippocampus, it should be noted that this adenosine may also play certain roles in the neuroprotective effect of AGE [51]. In addition, previous studies have reported that aqueous extract of garlic promoted adenosine signalling in the adenosine system of rat cardiac injury [52]. The dysfunction or damage of presynaptic cholinergic, glutamatergic, and GABAergic markers in the hippocampus was related to memory impairment in the Aβ-induced rats [53,54]. We found that administration of Aβ (1–42) into the lateral ventricle caused deficits in working and reference spatial memory, as evaluated by a RAM task. This is consistent with previous reports of Aβ-injected rats showing significant deficits in spatial cognition in the Morris water maze (MWM) [36], Y-maze [20], and a RAM task [55,56]. Aβ deposition might differentially affect excitatory and inhibitory synapses and cells, producing complex imbalances in circuit and network activity [57]. The present study demonstrated that all doses of AGE can protect against neurotoxicity caused by Aβ, as demonstrated by improvements in working memory. Although AGE did not significantly enhance reference memory, it did have a tendency to restore it. It has been suggested that AGE could improve working and reference memory as demonstrated through the use of RAM tasks. Moreover, AGE at doses of 250 and 500 mg/kg were able to protect against neurotoxicity caused by Aβ induction, as demonstrated by improvements in short-term spatial memory by MWM [58]. This is consistent with previous study of AGE treated rats showing ameliorate deficits in both short term and long term aspects of spatial memory as evaluated by MWM performance in senescence-accelerated mice [29]. AGE has also been shown to improve recognition memory, as evaluated by novel objective recognition in Aβ-induced rats [28]. Since working or short-term memory is a critical cognitive system used when remembering objects or places during goal-directed behaviour. Reference or long-term memory is required for temporally stable memories of those objects or places [59]. Furthermore, preliminary studies on pre-treatment of AGE at doses of 125, 250, and 500 mg/kg BW for 30 days. The results of RAM showed that all AGE-treated groups tended to remember the spatial location of short- term and long-term spatial memory better than the normal control. Moreover, all AGE doses significantly increased short-term and long-term memory (*p* *<* 0.05) by using MWM (data not shown). Therefore, it is possible that AGE may be effective in promoting the spatial memory in rats. 

## 5. Conclusions 

Our results demonstrated that AGE improved the working and reference spatial memory, by using RAM tests and is associated with increases in markers of VGLU1 and GAD proteins and ChAT-immunoreactive cells in the hippocampus of rat with Aβ-induced neurotoxicity. Therefore, we conclude that there may be health benefits associated with the consumption of aged garlic.

## Abbreviations

The following abbreviations are used in this manuscript:

ACh	Acetylcholine
AD	Alzheimer’s disease
AGE	Aged garlic extract
ANOVA	Analysis of variance
AP	Anterior-posterior
Aβ	β-amyloid
BW	Body weight
Ca^2+^	Calcium
ChAT	Choline acetyltransferase
CRD-HHP	Center for Research and Development of Herbal Health Products
DPX	Depex mounting medium
GABA	Gamma-aminobutyric acid
GAD	Glutamate decarboxylase
GAPDH	Glyceraldehyde 3 phosphate dehydrogenase
H_2_O_2_	Hydrogen peroxide
HCL	Hydrochloric
HPLC	High Performance Liquid Chromatography
MWM	Morri water maze
PBS	Phosphate buffer saline
pH	Potential of Hydrogen ion
RAM	Radial arm maze
ROS	Reactive oxygen species
S.E.M.	Standard error of mean
SAC	S-allyl cysteine
SDS-PAGE	Sodium dodecyl sulfate polyacrylamide gel electrophoresis
SI	Superior-inferior
TBS-T	Tris-buffered saline containing 0.1% tween 20
VGLUT1	Vesicular glutamate transporter1
VGLUT2	Vesicular glutamate transporter2
